# Belladonna in Hooping Cough

**Published:** 1855-12

**Authors:** 


*Belladonna in Hooping Cough.*

Dr. Turnbull makes the following remarks on the use of belladonna in hooping cough, in the *Medical Examiner* for August, which will be read with interest at a time when this refractory affection is prevailing as an epidemic :

The following was the method followed : The systembeing prepared by reducing the inflammation by the means before spoken of, obtain if possible, English extract of belladonna, fresh and good ; let the extract be triturated with water or simple syrup ; if it is to be kept for some time, add a small quantity of alcohol. The dose for a child three months old is the sixteenth of a grain every three hours, to a child one year one eighth of a grain, and so to other ages in proportion.

Inform the parent or nurse of the change it will produce upon the eyes, also that it may redden the skin. When full dilatation of the pupil is brought about, the medicine is to be intermitted till it has gone off again; the balladonna is to be administered in slightly increasing doses, so as to keep the child under its influence for several days or till the paroxysms are checked, which will usually occur toward the sixth or eight day of the second stage.

In the twenty cases cured by the use of the balladonna the cough and hoop returned in a few cases on exposure to cold or in disagreeable, windy weather ; but by combining the extract with syrup of ipecacuanha a few doses soon checked the cough and hoop ; in only one case out of this number was it complicated with inflammation of the lungs, and this case recovered.

The average duration of my twenty cases was ten days, after the hoop had commenced when the case was free from complications, which shows the great advantage of this treatment. The ordinary duration of the disease, when treated in the usual manner, is from 1½ to 3½ months; even by prussic acid, or the application of nitrate of silver, the average given is from two to three weeks. It is stated by Gibb that with the use of nitric acid, the average duration was only six or seven days. Several physicians who have used this remedy, however, do not find such favorable results from its use.

I have added to my communication some extracts from the ex perience of a few distinguished medical men on the use of thi important agent, belladonna.

This remedy was used in hooping cough about the year 1783 by Dr. Buckhaave, of Copenhagen, who gave the powdered root in doses of two grains, morning and evening, to a child of five or six years of age. The cure, it is stated, was generally accomplished in from seven to fourteen days.*

Dr. Miquel, of Neuerhaus, says the belladonna is a remedy upon which he can always depend in this disease. In the course of many epidemics which he has observed during fifteen years, he has constantly cured the cough in eight days.†

Dr. Samuel Jackson, of this city, late of Northumberland, who, although he was not the first to employ the belladonna, yet by his valuable publication in 1834 brought its virtues prominently before the medical public, has continued its use for twenty years, and his confidence in its powers to arrest the paroxysm and cure the second stage of hooping cough in the great majority of cases is undiminished.

Dr. Hiram Corson, formerly President of the Medical Society of the State of Pensylvannia, a distinguished practitioner of Montgomery county, Pa., in a paper on the efficacy of belladonna as a remedy in Pertussis, published in the American Journal of Medical Science, for October 1852, makes the following observations: "My experience in pertussis had satisfied me that of all the remedies in common use, those recommended by writers upon diseases of children are almost useless. Children affected in the winter continued to cough and strangle and suffer for many weeks with scarcely a perceptible amendment. It was painful to visit and mortifiying to prescribe for those afflicted with this malady."

He commenced the use of belladonna in four cases, and in one week they were all well. His method of using it was to begin with the sixteenth of a grain to children under one year every two hours, and increasing a little every day till full dilatation of the pupil occurred, the skin become flushed and vision confused; this

---

* Duncan's Commentaries for 1793, and Dr. Gibb on Pertussis, p. 282. 1854.

† Vol. vii. American Journal of Medical Sciences, p. 524, from Archieves Generales, August, 1830.

he accomplished by disolving eight grains of the extract in an ounce of water, nine drops of which contained the eighth of a grain.

In an epidemic in 1840 he used the belladonna in hundreds of cases with great relief in nearly all. By giving it in small doses at first, the fears of the patients were allayed. In 1847-8 he also prescribed it in numerous cases with much success. He concludes his paper in these words : "During the last seventeen years I have given the extract of belladonna to hundreds of patients from two months to fifteen years of age, and am firmly convinced that it has a greater control over hooping than any other remedy in common use. That while, in a few cases, the system did not seem susceptible to its action in the doses I have prescribed, yet in nearly all the disease yielded quickly. It is a safe and efficient remedy for pertussis in children of any age."

Dr. Eberle, in his Treatise on the Diseases of Children, second edition, remarks "that the balladonna has been highly celebrated, and is without doubt, by far, the best article of the kind we possess. My own experience leads me to testify confidently on this point. I have prescribed it within the last six years, (1834), in perhaps twenty cases, and in the majority of them with evident advantage." Prof. Borda, he remarks, was the first, he believed, who used it as a remedy, and his belief in its efficacy is almost unlimited.

Hufeland and Albert are almost equally decided in their praise of the virtues of this article.

The mortality from this disease in our city in 1850 was 114, 1852 168; and for 1853, was 64. In 1853 in the district of Richmond it occurred as an epidemic. In severe cases Dr. Janvier used the belladonna with the best results. "It mitigates the paroxysms better than any other sedative."*

Dr. Condie remarks in his work on diseases of children, that the narcotic from which the greatest amount of benefit is to be anticipated by this disease, is unquestionably the belladonna ; it has been very extensively employed, and the evidence in its favor is strong and conclusive, (by Kahliss, Janin, Hufeland, Widemann, Raisin, Guibert, Albert. Shafer, Laennec, Muller, Blache, Maunsell. and Lombard )

He further remarks that he had given the belladonna a very fair trial, and has, in many cases, been pleased with the prompt and decided relief produced by it, while in other instances it appeared to exert no influence whatever.

I think that this last remark may be often accounted for by the

---

* Report Phila. County Med. Society for 1853.

bad character of the belladonna, which is even found in some of the drug stores of this city, for it is an uncertain preparation unless when procured by evaporation in vacuo, for some samples from some of the Parisian shops were found by Orfila to be quite inert.

Dr. Williams, of London, has used belladonna with great advatage in his practice. He gives it in a quarter grain doses to a child of two years, increasing the doses to double that quantity or more where it fails to relieve. He remarks that these doses, in general, cause some dilatation of the pupil, and conceives that the radical agency of the drug depends on the same power to diminish irritability of the bronchial and laryngeal muscles which is here evinced with regard to the iris.†

Dr. G. A. Rees has found belladonna one of the most efficioacus remedies in pertussis.‡

Dr. Waller cured two cases with the twelfth of a grain of extract three times a day; prussic acid and conium had failed in affording any permanent relief.§

Aberle assigns the highest place among narcoties to be belladonna in hooping cough.

Dr. Churchill says that this is perhaps the most influential narcotic and sedative we possess (in pertussis); it has been very extensively employed and the evidence in its favor is very strong.||

Belladonna has been eminently useful in the epidemics of hooping cough which M. Debreyne has observed, but recourse should not be had to it till the inflammatory element has been overcome by leeches, emetics, etc.

Dr. A. T. Thompson says: "I have ordered the extract of belladonna in doses of one-eight of a grain to a child of eight years, and gradually increased the dose to a quarter of a grain. Its power over the cough is extraordinary."¶

† Gibb on hooping cough, p. 284, from Medical Gazette, Feb. 1838.

‡ Diseases of Children, second edition, 1844.

§ Lancet, Vol. i. 1842, p. 137.

|| Elements of Materia Medica.

¶ London Journal of Medicine, April, 1850.

